# Effect of Energy Density on Mechanical Properties of NiTiCu Shape Memory Alloys Prepared by SLM

**DOI:** 10.3390/ma17235693

**Published:** 2024-11-21

**Authors:** Yi Ba, Yan Lv, Miaoning Yan, Hongxu Jin, Liang Guo, Qingmao Zhang

**Affiliations:** 1 Guangdong Provincial Key Laboratory of Nanophotonic Functional Materials and Devices, School of Optoelectronic Science and Engineering, South China Normal University, Guangzhou 510006, China; 2022010151@m.scnu.edu.cn (Y.B.); scut@foxmail.com (Y.L.); 2 Riton Additive Technology Co., Ltd., Guangzhou 510320, China; 3 College of Physics and Optoelectronic Engineering, Jinan University, Guangzhou 510632, China

**Keywords:** laser selective melting, NiTi shape memory alloy, microstructure, mechanical properties

## Abstract

In the Ni-Ti shape memory alloy system, Cu elements are used to replace Ni elements. A NiTiCu alloy with a molar ratio of 45:50:5 was prepared using laser selective melting technology. The density, composition, microstructure, and mechanical properties of the NiTiCu alloy were investigated. The results indicate that the highest density, exceeding 99.7%, was achieved when processing NiTiCu with parameters of 115 mm/s and 90 W. X-ray diffraction (XRD) analysis revealed that the primary phases of the sample are B2 and a minor amount of NiTi0.8Cu0.2. Energy-dispersive X-ray spectroscopy (EDS) observations of the NiTiCu alloy in the X-Y and X-Z planes show that Ni, Ti, and Cu elements are distributed nearly uniformly. Electron backscatter diffraction (EBSD) analysis revealed fine grain sizes, with grain sizes ranging from 140 μm to 160 μm. The X-Y plane predominantly exhibits equiaxed grains with a grain orientation between <111> and <101>, and a texture strength of 1.312; the X-Z plane predominantly exhibits columnar grains with grain orientations between <001> and <101>, and a texture strength of 1.427. The sample demonstrates good mechanical properties at room temperature, with a tensile strength of 375 MPa, exhibiting a ductile–brittle mixed fracture mode. The average microhardness is 240 HV for the X-Y plane and 235 HV for the X-Z plane.

## 1. Introduction

For a long time, NiTi alloys have been extensively used in medical and industrial fields due to their excellent mechanical properties, biocompatibility, and corrosion resistance [1,2,3,4]. Over the past decade, the enhancement of the mechanical properties of NiTi alloys through the addition of Cu elements has become a significant research focus. The incorporation of Cu reduces the Ti3Ni4 precipitate phase and promotes the formation of twinned phases, leading to improved mechanical performance of NiTi alloys [5,6,7]. However, traditional machining methods for NiTiCu alloys, such as milling, turning, and welding, often face challenges such as burr formation, low precision, and tool adhesion. Conventional processes such as melting–rolling, wiredrawing, machining, and welding are typically limited to producing simple shapes like rods, wires, and plates, making it difficult to fabricate components with complex shapes or three-dimensional structures. This limitation significantly hampers the further development and application of NiTi alloys [8,9,10,11,12].

Additive manufacturing (AM), also known as three-dimensional (3D) printing, involves the production of parts directly from a 3D digital model through a layer-by-layer process, allowing for the creation of components with complex geometries and internal features [13,14]. To meet the demands of modern industry for complex geometries, technologies such as Directed Energy Deposition (DED), Selective Laser Melting (SLM), and Electron Beam Melting (EBM) are employed. However, it has been reported in the literature that the mechanical properties of NiTiCu produced via additive manufacturing are often inferior, achieving only about 70% of those of conventional NiTiCu alloys, which severely limits practical applications [15,16,17,18]. Research indicates that additive manufacturing can introduce relevant defects in metals, such as residual stresses, cracks, and voids. Under tensile stress, NiTiCu can exhibit significant stress concentrations and premature brittle fracture. To address these issues, researchers have conducted the following studies.

Sun Dong et al. [19] conducted annealing treatments on NiTiCu at temperatures of 300 °C, 450 °C, and 600 °C. Nanotwins were observed in the NiTiCu samples annealed at 300 °C, while a significant number of twin variants emerged in the samples annealed at 450 °C and 600 °C. Nanotwins contribute to enhancing the material’s strength and suppress the formation of martensitic variants. Under compression loading, the NiTiCu alloy treated at 300 °C exhibits superior mechanical properties. The application of heat treatment to the material reduces defects, induces solid solution strengthening, and introduces twin strengthening. However, annealing increases the grain size of the material, which precludes the development of grain size strengthening and extends production time, making it unsuitable for large-scale manufacturing.

Berk Keskin et al. [20] employed the Self-Propagating Combustion Method to synthesize a NiTiCu alloy with a molar ratio of 40:50:10, preheating the printing substrate at temperatures of 230 °C, 320 °C, and 410 °C prior to the fabrication process. X-ray diffraction (XRD) analysis revealed that the samples predominantly consist of the B2 phase with minor Ti2Ni precipitates. Electron backscatter diffraction (EBSD) and transmission electron microscopy (TEM) observations showed that, apart from minor martensite, Ti2Ni(Cu), and Ni(Cu)2Ti–Ni(Cu)3Ti phases, the primary phase is the B2 crystal structure. TEM analysis also identified a small amount of monoclinic martensite (B19′) and a significant presence of twin structures in the samples. Although preheating the printing substrates can yield high-quality NiTiCu alloys, it is dependent on the use of specific equipment.

Francesca Villa et al. [21] utilized a casting method to fabricate a NiTiCu20 alloy and investigated the microstructural characteristics and the evolution of secondary phases. The samples demonstrated excellent wear resistance at room temperature. Scanning electron microscopy (SEM) observations revealed the presence of nickel-rich and nickel-poor regions within the samples. Analysis confirmed the expected phases of B19, cubic Ti2Ni, and residual B2 at room temperature.

Kireeva et al. [17] synthesized a NiTiCu10 alloy through a melting process, which exhibited a maximum tensile strength of 250 MPa and a grain orientation of [011], with a maximum tensile elongation of 7.14%. During mechanical loading, phase transformations from B2 to B19 to B19′ occurred within the alloy, and a significant amount of twinning was observed. These phenomena are beneficial to the material’s performance.

WS Ko et al. [22] employed thermodynamic integration methods and molecular dynamics simulations to calculate the properties of Ni50Ti50, Ni50Ti48Cu2, Ni50Ti46Cu4, Ni50Ti44Cu6, Ni50Ti42Cu8, and Ni50Ti40Cu10 alloys. They achieved an atomic-level understanding of the phase transformation behavior of NiTiCu shape memory alloys. Free energy calculations based on the thermodynamic integration method indicated that both composition and grain size can be adjusted to achieve minimal hysteresis. However, significant effects of increased Cu content were only observed when phase transformation modes were altered at relatively lower Cu concentrations.

Long C et al. [23] fabricated a NiTiCu alloy with a copper content of 20.80% using arc additive manufacturing techniques. The results demonstrated that the NiTiCu alloy exhibited a well-defined morphology, with microstructures including columnar, equiaxed, and needle-like particles. The material predominantly exhibited the martensitic B19 structure. The tensile strength was approximately 232 MPa, and the fracture strain was around 3.72%.

Current research typically involves substituting Ni with Cu in the range of 10 to 20 at%. Various studies have investigated the effects of Cu content on martensitic transformation pathways, microstructure, atomic positions, and functional performance, as well as improvements relative to the NiTi system. However, previous studies have primarily focused on the impact of copper addition on the properties of shape memory alloys, while the influence of energy density on the addition of copper has not been clearly investigated. Although extensive research has shown that substituting nickel with copper can indeed enhance the toughness and plasticity of NiTi alloys, the low abundance of copper compared to nickel in the Earth’s crust raises concerns about sustainability when using large amounts of copper as a substitute. Furthermore, substituting copper for nickel inevitably increases the overall cost of NiTiCu alloys. To improve the Poisson’s ratio of NiTi shape memory alloys while reducing the amount of metallic copper used, this study posits that the addition of 5% copper can increase the Poisson’s ratio by 0.004. However, increasing the copper content to 10% would result in a decrease in the Poisson’s ratio. Therefore, this research will use 5% copper as a substitute for nickel, focusing on the effects of energy density on the microstructure and mechanical properties of NiTiCu alloys containing 5% copper.

## 2. Materials and Methods

### 2.1. First Principles Calculation

In this paper, relevant theoretical calculations were performed using the Quantum ESPRESSO 7.2 software package. The calculations were conducted in the following sequence: constructing a structural model to obtain actual unit cell parameters and volume parameters, applying deformation to the unit cell, incorporating square deformation, and setting triangular deformation. Considering the isotropic deformation of the body-centered cubic (BCC) lattice structure of the material, which corresponds to uniform stretching of the lattice vectors ${a’}_{i}=(1+\eta )a_{i}$, (i = 1, 2, 3, …), the elastic behavior of the BCC crystal structure can be described by three independent elastic constants: C11, C12, and C44. By applying an isotropic deformation (i.e., uniformly stretching or compressing the lattice in all directions), the combination of C11 and C12 can be determined. This was achieved by altering the lattice parameter a, calculating the total energy difference before and after deformation, and then solving for C11 + 2C12 using the appropriate formula.

(1) $$U-U_{0}=\frac{3}{2}Ω(C11+2C12)\eta^{2},$$

Wherein U and U_0_ represent the total energies after and before deformation, respectively; Ω denotes the unit cell volume; and η signifies the deformation parameter. Subsequently, by considering the addition of a square deformation, the following relationship can be obtained:

(2) $$U-U_{0}=3Ω(C11-C12)\eta^{2}.$$

The square deformation of a unit cell can be achieved by considering an expansion (1 + η) along the x and y axes and a contraction (1 − 2η) along the z axis. By combining Equations (1) and (2), C11 and C12 can be solved. Finally, through a triangular deformation, C44 can be calculated according to Equation (3):

(3) $$U-U_{0}=\frac{1}{2}ΩC44\eta^{2}.$$

Poisson’s ratio is an elastic constant that reflects the lateral deformation of a material, commonly used to describe the uniaxial tensile or compressive properties of materials [24,25]. It serves as an indicator for characterizing both the elasticity and plasticity of materials; a higher Poisson’s ratio suggests better elasticity and plasticity of the material. The formula for calculating Poisson’s ratio is as follows:

(4) $$v=\frac{3B_{H}-C11+C12}{2(3B_{H}+C11-C12)}$$

In this calculation, three Cu element proportions were considered, with the specific proportions and calculated results of Poisson’s ratio presented in Table 1. When the atomic ratio of Cu is 5%, the Poisson’s ratio reaches its highest value, indicating optimal elasticity and plasticity. Therefore, in this experiment, NiTi alloy doped with 5% Cu will be studied.

### 2.2. Experimental Material

**Figure 1:**
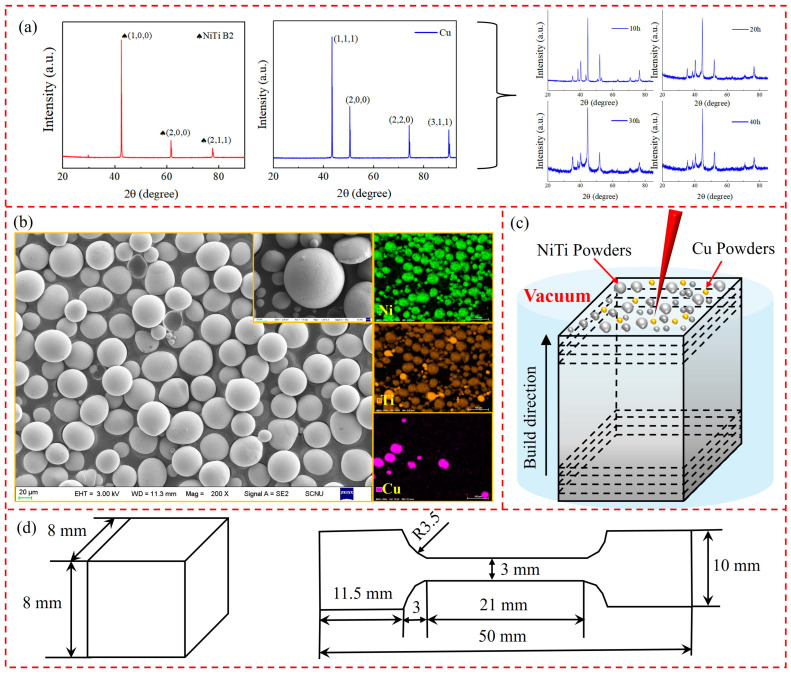
(**a**) XRD pattern of NiTi powders; (**b**) SEM micrograph and corresponding elemental SEM-EDS map; (**c**) schematic diagram of 3D printing; (**d**) print sample of NiTi alloy.

In this study, NiTi powders with a particle size range of 15 to 53 μm and Cu powders were used. The NiTi and Cu powders were mixed and ball-milled for 10, 20, 30, and 40 h, respectively. The resulting NiTiCu powders were then dried in a vacuum oven with a drying process set at 80 °C for 2 h. The prepared powders were characterized using X-ray diffraction (XRD), as shown in Figure 1a. The NiTiCu powder milled for 20 h was selected for further analysis, as depicted in Figure 1b. It can be observed that both NiTi and Cu powders exhibited good sphericity and smooth surfaces, with Cu particles evenly distributed in the powder mixture. The laser used in the experiment was the D150 laser, developed and manufactured by Ruitong Company, with a wavelength of 1024 nm. Laser powder bed fusion was performed with synchronized powder feeding. During the process, argon gas was used for protection. NiTiCu alloys were fabricated in an argon environment with an oxygen content of less than 0.01% and an energy density of 40.3 J/mm^3^ to 83.33 J/mm^3^. The schematic diagram of 3D printing is shown in Figure 1c, while the printed samples are depicted in Figure 1d. The samples consist of a cube measuring 8 mm × 8 mm × 8 mm and a tensile specimen measuring 10 mm × 50 mm, designated for subsequent research and testing. The printing parameters are outlined in Table 2.

### 2.3. Detection Method

This study utilized systematic testing equipment and methods to analyze the microstructure and mechanical properties of the materials. Density measurements were conducted using Archimedes’ principle with 100% anhydrous ethanol as the liquid medium. Phase identification was performed using an X-ray diffractometer BRUKER D8 ADVANCE DAVINCI (Bruker Corporation, Karlsruhe, Germany). The NiTiCu samples were sequentially polished using sandpapers of grit sizes 200#, 600#, 1000#, 1500#, and 2000#, followed by electrolytic polishing treatment with an electrolyte solution consisting of 25% H_2_SO_4_ and 75% CH_3_OH. Subsequently, Electron Backscatter Diffraction (EBSD) imaging was conducted using a ZEISS Sigma300 (Carl Zeiss AG, Oberkochen, Germany). The tensile strength of the NiTi alloy was evaluated at room temperature using a universal tensile testing machine Roell 1484 (Zwick, Ulm, Germany), with a total of three samples being tested and the average value taken. The tensile fracture morphologies were examined using a scanning electron microscope ZEISS Gemini 500 (Carl Zeiss, Oberkochen, Germany). Microhardness measurements in both the X-Y and X-Z directions were performed using a Buehler VH1202 (Wilson, Chicago, IL, USA) microhardness tester, with a pressure standard of HV0.2 and a dwell time of 5 s.

## 3. Results

### 3.1. Density and Phase

**Figure 2:**
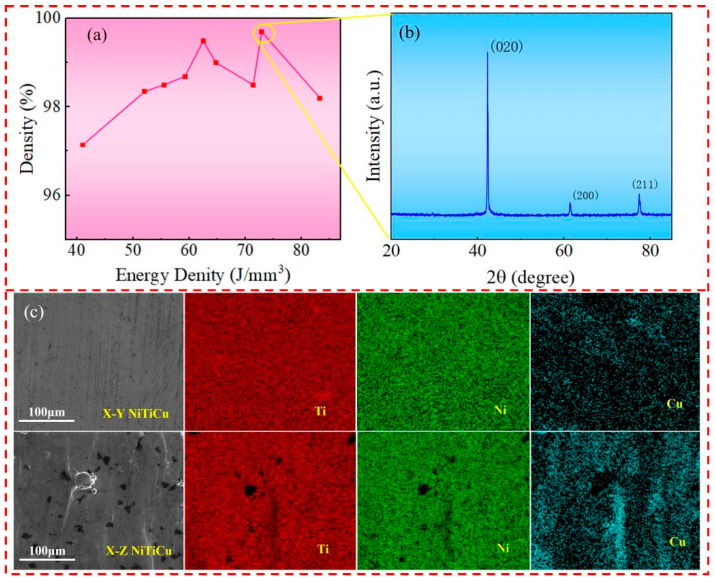
(**a**) Density of NiTiCu alloy; (**b**) XRD of NiTiCu alloy prepared at an energy density of 73 J/mm^3^; (**c**) EDS of NiTiCu alloy.

Figure 2a illustrates the relative density of NiTiCu samples prepared using different energy densities. The data indicate that as the laser energy density increases, the relative density first increases and then decreases, with the highest relative density of 99.7% achieved at an energy density of 73 J/mm^3^. This trend is likely due to the fact that at low energy densities, the molten pool temperature is insufficient, leading to poor flowability and the formation of irregular voids. Conversely, excessively high energy densities can cause Ni evaporation, resulting in voids and cracks [26].

The X-ray diffraction (XRD) analysis of NiTiCu samples prepared at an energy density of 73 J/mm^3^ is shown in Figure 2b. The XRD pattern reveals peaks at (020), (200), and (211), with the (020) peak being the most prominent. A comparison with Figure 1b shows a shift in the dominant peak within the 2θ angle range of 42.0° to 43.5°, indicating lattice distortion during the Selective Laser Melting (SLM) process [27].

Figure 2c displays the Energy Dispersive Spectroscopy (EDS) analysis of the X-Y and X-Z planes of the SLM-NiTiCu samples, revealing the elemental distribution. Based on the pre-treatment of the powders, the distribution of Ni, Ti, and Cu elements appears to be relatively uniform.

### 3.2. Grain Orientation and Grain Boundary Characteristic

**Figure 3:**
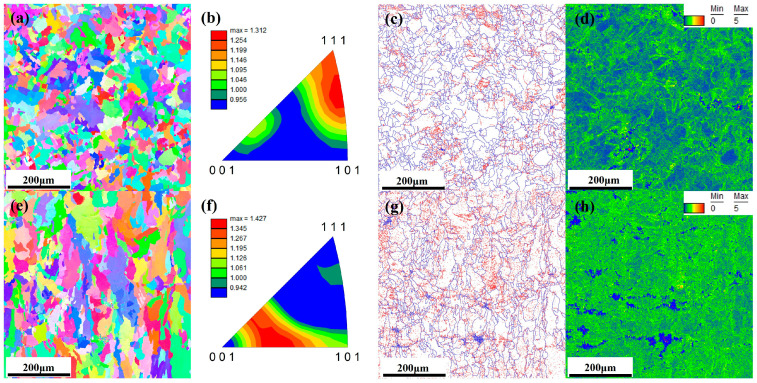
(**a**–**d**) X-Y plane of NiTiCu alloy: pole figure; inverse pole figure; angle boundary map; kernel average misorientation (KAM) map; (**e**–**h**) X-Z plane of NiTiCu alloy: pole figure; inverse pole figure; angle boundary map; kernel average misorientation (KAM) map.

The grain distribution in both X-Y and X-Z planes of NiTiCu samples prepared using a parameter of 73 J/mm^3^ was observed via Electron Backscatter Diffraction (EBSD), with the results presented in Figure 3. Figure 3a,e present the inverse pole figures (IPF) for NiTiCu in the X-Y and X-Z planes, respectively. In the X-Y plane, the grains are predominantly equiaxed. The X-Z plane, however, consists of a small amount of equiaxed grains and a large number of columnar grains, with the columnar grains growing parallel to the build direction, which is also the direction of heat supply. The average grain size in both the X-Y and X-Z planes ranges between 140 μm and 160 μm, with the grains in the X-Y direction being finer. This is attributed to the remelting and temperature gradients during the Selective Laser Melting (SLM) process. Remelting is more pronounced in the X-Y plane compared to the X-Z plane. In the X-Z plane, deeper molten pools experience more extensive remelting, and the laser energy decreases with depth, which hinders grain refinement. Additionally, the bottom of the molten pool, being closer to the cooling baseplate, cools faster, which promotes the growth of columnar grains [28,29]. Research by Guo et al. [30,31] indicates that during additive manufacturing, regions with lower cooling rates are more likely to produce finer grains. This phenomenon is commonly observed in cubic alloys processed through additive manufacturing [32,33,34].

According to Yuan et al. [35], the prepared NiTi alloy exhibited a significant <001> texture orientation, with a maximum texture strength of 7.4. Figure 3b,f present the inverse pole figures (IPFs) for the X-Y and X-Z planes, respectively. The grain orientation in the X-Y plane shows a texture strength of 1.312 between <111> and <101>, while in the X-Z plane, the texture strength is 1.427 between <001> and <101>. The observed decrease in texture strength is advantageous for enhancing tensile properties.

Figure 3c,g display the EBSD grain boundary maps for the X-Y and X-Z planes. The blue and red lines denote large-angle grain boundaries (LAGBs) and small-angle grain boundaries (SAGBs, 2° to 15°), respectively. The proportion of SAGBs in the X-Y and X-Z planes are 37% and 55.3%, respectively. This observation is corroborated by the kernel average misorientation (KAM) maps shown in Figure 3d,h. The KAM values are lower in the blue regions and higher in the green regions.

Research has indicated that the transition from large-angle grain boundaries to small-angle grain boundaries is associated with dynamic recovery (DRV) and dynamic recrystallization (DRX) during the additive manufacturing process [32]. The temperature gradients and solidification rates in additive manufacturing lead to the formation of high-density dislocation networks, which evolve into dislocation walls [36]. These dislocation walls further develop into small-angle grain boundaries, resulting in subgrains, which is indicative of dynamic recovery. When internal stress accumulates to a critical level, small-angle dislocations continue to absorb additional dislocations, leading to dynamic recrystallization and the formation of large-angle grain boundaries. This process significantly consumes dislocations and contributes to grain refinement.

### 3.3. Tensile Strength

**Figure 4:**
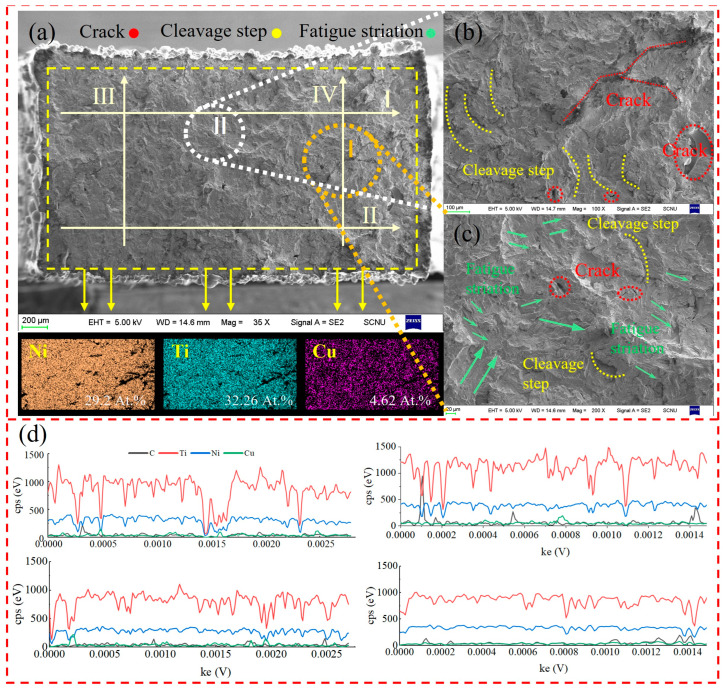
Tensile of NiTiCu alloy; (**a**) tensile fracture; (**b**) Region I; (**c**) Region II; (**d**) EDS line scanning of (I–IV) in Figure 4a.

Through tensile testing, the tensile strength of the SLM-NiTiCu shape memory alloy prepared using a parameter of 73 J/mm^3^ was found to be 375 MPa. The fracture surface is shown in Figure 4a, which displays distinct river-like patterns, indicative of a mixed brittle–ductile fracture mode. Magnified views of regions I and II reveal numerous cleavage planes and irregular voids and cracks. Therefore, it can be confirmed that the tensile fracture is characterized by a combination of brittle and ductile features. The observed voids and cracks at the fracture surface are indicative of points of failure under tensile stress and are one of the contributing factors to the tensile fracture of NiTiCu.

Energy Dispersive Spectroscopy (EDS) surface scanning of the tensile fracture of the NiTiCu alloy revealed the presence of Ni, Ti, and Cu elements at the fracture site. EDS line scans were conducted along the four lines marked as I, II, III, and IV in Figure 4a. The results, shown in Figure 4d, indicate that the Ti content at the fracture site is consistently higher than that of Ni in all four line scans. The primary phase is Ti2Ni, with a minor presence of NiTi0.8Cu0.2.

Studies have indicated that under tensile stress, the brittle Ti2Ni phase may experience delamination, with fractures occurring near regions containing 80% Ti2Ni [30]. As shown in Figure 4b, the crack features are primarily attributed to stress concentration, with secondary cracks providing additional stress concentration points, facilitating easier crack propagation. The substantial difference in thermal expansion coefficients between the matrix phase and the intermetallic phases may also contribute to the formation of microcracks during the cooling process. Furthermore, the mechanical properties of the NiTiCu alloy are influenced by the grain size and the nature of the precipitates. According to the Hall–Petch relationship, smaller grain sizes can enhance both strength and elongation [31]. Fine and uniformly distributed precipitates produce a strain-hardening effect, strengthening the material. However, irregular and coarse precipitates are prone to crack initiation and propagation, leading to a reduction in strength and ductility. One of the factors contributing to the poor tensile performance of the material is the presence of the brittle Ti2Ni phase. Additionally, columnar grains may break along the grain boundaries.

### 3.4. Microhardness

**Figure 5:**
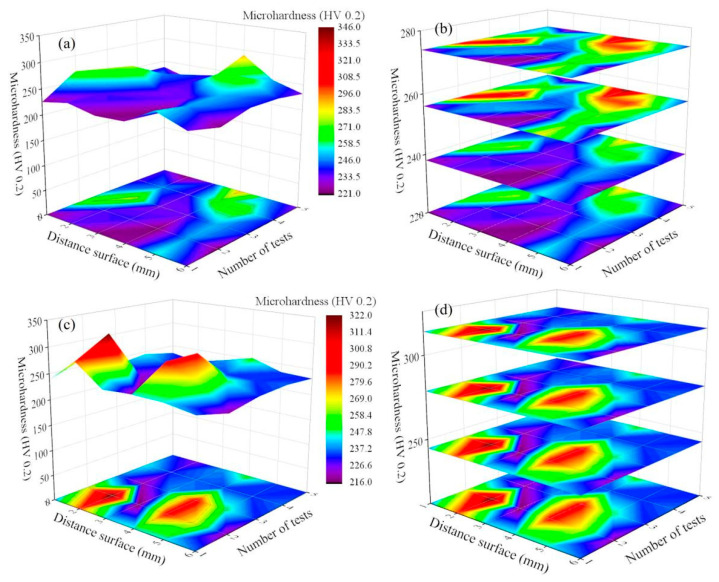
Microhardness of NiTiCu alloy (a,**b**), X-Y plane microhardness; (**c**,**d**) X-Z plane microhardness.

The microhardness distribution in both the X-Y and X-Z planes of the 73 J/mm^3^ SLM-NiTiCu is illustrated in Figure 5. The results indicate that the microhardness of NiTiCu alloy ranges from 225 to 270 HV0.2, which is relatively high compared to reported values in the literature (201–245 HV [37], 220 HV [38], 225 HV [39], 200–260 HV [40], 238 ± 4 HV, and 235 HV [41]). The primary reason for the increased microhardness of NiTiCu is the solid solution strengthening effect achieved by the addition of Cu. Additionally, the formation of equiaxed grains in the cladding layer effectively enhances the microhardness of the NiTi alloy. Notably, the microhardness in the X-Y plane is higher than that in the X-Z plane. This is because the columnar grains in the X-Z plane have smaller grain boundaries and their resistance to external deformation is lower compared to equiaxed grains. According to the Hall–Petch relationship [42], coarser grains reduce the material’s ability to resist external deformation, which is detrimental to increasing the microhardness. As can be clearly observed from Figure 3a,e, the X-Y direction has more equiaxed grains, leading to higher microhardness. An energy density of 73 J/mm^3^ can result in a more uniform phase distribution and smaller grain sizes. The addition of 5% copper can enhance hardness, contributing to the improvement of mechanical properties in NiTiCu alloys.

## 4. Conclusions

In this study, NiTiCu alloy was prepared by laser powder bed fusion using nine sets of process parameters with energy densities ranging from 40.3 J/mm^3^ to 83.33 J/mm^3^. The results indicated that the best density, which reached 99.7%, was achieved when using an energy density of 73 J/mm^3^. The X-ray diffraction analysis revealed three primary peaks, (020), (200), and (211), with the (020) peak being the dominant one, indicating a predominantly BCC (body-centered cubic) structure. In the X-Y and X-Z planes, the primary grain types are equiaxed grains and columnar grains, with small-angle grain boundary fractions of 37% and 55.3%, respectively. The texture strengths are 1.312 between <111> and <101> in the X-Y direction and 1.427 between <001> and <101> in the X-Z direction.

In terms of mechanical properties, the tensile strength of the alloy is 375 MPa. The fracture surface exhibits a few cracks and voids. EDS analysis of the fracture site identifies Ti2Ni as the primary brittle phase. During tensile testing, Ti2Ni, along with cracks and voids, serves as initiation points for fracture. Microhardness testing indicates that the microhardness of the NiTiCu alloy ranges from 225 to 270 HV0.2, reflecting relatively high hardness. This study confirms the feasibility of preparing NiTiCu alloys using laser selective melting technology and establishes a foundation for the efficient production of NiTiCu-based ternary shape memory alloys using this method.

## Figures and Tables

**Table 1:** Atomic ratios and calculated results of NiTi shape memory alloy.

Sample	Ti (at.%)	Ni (at.%)	Cu (at.%)	Poisson’s Ratio
1	50.00	50.00	0.00	0.222
2	50.00	45.00	5.00	0.226
3	50.00	40.00	10.00	0.215

**Table 2:** Process parameters for SLM NiTiCu shape memory alloy.

Sample	Scanning Speed (mm/s)	Laser Power (w)	Energy Density (J/mm^3^)
1	700	100	59.52
2	700	120	71.42
3	700	140	83.33
4	800	100	52.08
5	800	120	62.5
6	800	140	73
7	900	100	40.3
8	900	120	55.56
9	900	140	64.81

## Data Availability

The original contributions presented in the study are included in the article, further inquiries can be directed to the corresponding authors.
